# Plants Used as Anticancer Agents in the Ethiopian Traditional Medical Practices: A Systematic Review

**DOI:** 10.1155/2018/6274021

**Published:** 2018-10-03

**Authors:** Nigatu Tuasha, Beyene Petros, Zemede Asfaw

**Affiliations:** ^1^College of Natural and Computational Sciences, Mizan-Tepi University, P.O. Box 121, Tepi, Ethiopia; ^2^Addis Ababa University, Department of Microbial, Cellular and Molecular Biology, P.O. Box 1176, Addis Ababa, Ethiopia; ^3^Addis Ababa University, Department of Plant Biology and Biodiversity Management, P.O. Box 3434, Addis Ababa, Ethiopia

## Abstract

**Background:**

This systematic review aimed at examining the use patterns of Ethiopian anticancer traditional medicinal plants (MPs) in view of recommending further validation studies.

**Methods:**

The information was retrieved from PubMed according to the PRISMA guideline. The electronic library of Addis Ababa University and relevant church-based religious books were also inspected for additional data.

**Results:**

From 34 studies meeting specific inclusion/exclusion criteria, 119 anticancer MP species distributed in 98 genera and 57 families were recorded. Fabaceae (10.92 %) and Euphorbiaceae (10.08 %) were the most widely used families.* Plumbago zeylanica *(Plumbaginaceae) was the most frequently used anticancer MP species. Shrubs (42.02 %) and herbs (35.29 %) were dominant growth forms. About 89.08 % of the MPs were collected from wild habitats. Leaves (33.73 %) and roots (27.11 %) were the most frequently harvested parts. The most favored routes were dermal (33.33 %) and oral (29.25 %). About 87.07 % preparations were made from fresh plant materials. Breast cancer and skin cancer were treated with 14 % and 10.76 % of anticancer MPs, respectively.

**Conclusion:**

The review showed that anticancer MPs are widely used across the regions of Ethiopia. Most of the MPs are not scientifically experimented and yet are at a higher vulnerability to loss mainly by human activities. Calling for conservation measures, we recommend experimental validation of the frequently used anticancer MPs. This augments global anticancer drug researches.

## 1. Background

### 1.1. Cancer

Cancer is the name given to a group of diseases comprising a combination of genetic, metabolic, and signaling pathway aberrations [1]. It is usually a fatal disease, which constitutes an enormous burden on society in both economically developed and underdeveloped nations alike [2]. It is the second leading cause of death globally next to cardiovascular diseases; and available estimates by GLOBOCAN (an International Agency for Research on Cancer-IARC) show that about 14.1 million new cancer cases and 8.8 million deaths occurred in 2012 worldwide. Furthermore, by 2012 32.6 million people were living with cancer, within 5 years of diagnosis [3, 4]. Overall, 57 % of new cancer cases, 65 % of the cancer deaths, and 48 % of the 5-year prevalent cancer cases occurred in the less developed regions [3]. According to WHO estimates in 2015, about 200 known types of cancer exist and the most common causes of cancer deaths include cancer of the lung (1.69 million), liver (788, 000), colorectal (774, 000), stomach (754, 000), and breast (571, 000) [4]. The prevalence of cancer is increasing because of the growth and aging of the population, as well as an increasing prevalence of established risk factors such as smoking, overweight, physical inactivity, and changing reproductive patterns associated with urbanization and economic development [3, 5].

In Africa, cancer is alarmingly becoming a critical public health problem, with cancer forms attributed to infectious agents (e.g., cervical cancer, liver cancer, Kaposi sarcoma, and urinary bladder cancer) being the dominant types [6]. According to the IARC, about 715,000 new cancer cases and 542,000 cancer deaths occurred in 2008 in Africa [6, 7]. The numbers are projected to double by 2030 due to the aging and fast growth of the population. The potential of being even higher has been imagined because of the adoption of behaviors and lifestyles associated with economic development [6–9]. Thus, prostate cancer in men and breast cancer in women have now become the most commonly diagnosed cancers in some parts of Africa [3, 10].

Reports on the prevalence of cancer in Ethiopia are scanty and difficult to verify because oncology services are inadequate and national registry centers do not exist; there are no control and prevention programmes against the disease; diagnosis and treatment services are very limited [11–13]. However, the limited reports show that Kaposi sarcoma, liver, prostate, cervical, and breast cancer are the most common cancers [14] in Ethiopia. According to the cancer country profiles of WHO, most prevalent forms among males are colorectal, Kaposi sarcoma, leukemia, lymphomas (non-Hodgkin), and prostate cancers in the order of listing, whereas cancer of the breast, cervix-uteri, ovary, colorectal, and leukemia, in the order given, are topping the list among females [15]. A study at the radiotherapy center of Tikur Anbessa specialized hospital (currently the only one in the country) showed that breast cancer had a high prevalence (27.8 %) [16]. On the scale of mortality leukemia (12.7 %) in males and breast cancer (24.4 %) in females were reported to be the highest in 2014 [15].

### 1.2. Traditional Medicines and Cancer

Herbal medicine (phytomedicine, phytotherapy, or botanical medicine) is the oldest system of complementary and alternative medicine (CAM) in the world with a history of more than 2,000 years [17]. It is made exclusively from plants and is used by all societies and are common to numerous cultures [18]. There are, however, variations in the preparation and treatment procedures throughout the world [19]. Certain herbal medicines defend the body from malignancy by augmenting detoxification or cleaning the body. Some biological response modifiers derived from herbs are known to hinder the growth of cancer by modifying the activities of hormones and enzymes, while others diminish lethal side effects and complications of chemotherapy and radiotherapy [20].

The use of CAM is common among cancer patients in general and breast cancer patients, in particular [21]. They are perceived by the general public to be safe, cause less complications, and are less likely to cause dependency [19]. Most cancer patients combine herbal remedies with conventional therapy in the hope of boosting the effect of conventional medicine [22]. Nevertheless, cancer patients who use CAM report outcomes including improvement of clinical symptom, quality of life, reduction of chemo/radiotherapy induced side effects, and reduced tumor size [23–25]. Supporting conventional cancer treatment, preventing recurrence and eventual prolonging of survival were also reported [26]. In addition to these claims, anticancer agents derived from plants were shown to inhibit angiogenesis, suppress cell proliferation, inhibit or reverse tumor development, and show general antioncogenic effects [27]. A number of phytoconstituents resulting from the herbs were also reported to assist the body's immune system to combat cancer more efficiently [20].

### 1.3. Ethiopian Flora and Medicinal Plants

Historical accounts substantiate age-long usage of traditional medicines (TMs) in Ethiopia. For instance, collections of medicoreligious manuscripts of the Axumite kingdom, medical textbooks that have been written in Ge'ez and Arabic languages between mid-17^th^ and beginning of the 18^th^ century are few among long-standing sources [28, 29]. It is widely reported that over seventy percent of the Ethiopian people depend on TM for their healthcare, where more than 95 % of traditional medicines are sourced from plants [30, 31]. Ethiopia's location in a tropical area, its huge landmass, and incredible variation in altitude ranging from about 120 meters below sea level to 4,543 meters above sea level have strongly influenced the range of its ecosystem and have contributed to the high diversity and rate of endemism in the flora [32–34]. Thus, the flora of Ethiopia has about 6,027 species of vascular plants with 10 % endemism [35–38]. According to recent reports, over 1000 species are used as traditional medicinal plants (MPs) of which about 33 species are endemic to Ethiopia [39, 40].

## 2. The Rationale for the Systematic Review on the Anticancer MPs of Ethiopia

It is evident that the incidence of cancer is on the rise globally. The disparity in cancer survival between high and low economic settings mainly lies on two factors: the stage of its diagnosis and availability and access to treatments [6, 81, 82]. Although surgery is the existing standard curative therapy for most cancer forms, its therapeutic efficacy is compromised as most tumors are often diagnosed at an advanced stage, particularly in the underdeveloped part of the world [83, 84]. Therefore, treatments such as radiotherapy and chemotherapy are next ladder therapies although they achieve only very modest results [85]. The everincreasing occurrence of cancer and the severe side effects and limited efficacy of current cancer chemotherapy based on synthetic drugs shift the attention toward drugs of plant origin [86, 87].

In Ethiopia, environmental degradation, deforestation, intermittent drought, high rate of cultural and habitat loss, and various anthropogenic activities are threatening the MPs. However, recent ethnobotanical studies from different parts of the country have shown that TM is widely practiced in the country. It is also claimed that cancer patients prefer TMs to conventional therapeutic approaches mainly due to its cultural acceptance and ease of access [37, 39, 51, 52, 56, 59, 72, 73, 76, 77, 88–99]. The Ethiopian flora largely remained untouched with tremendous potential that could offer a lot in the fight against cancer. Therefore, it is believed that the systematic review of ethnobotanical studies reported from Ethiopia would help identify potential anticancer MPs. This will serve as a basis for initiating rigorous scientific investigations for the chemopreventive and anticancer attributes of the MP species most frequently used across the regions of Ethiopia. Such investigation would contribute to the global anticancer drug discovery effort.

## 3. Methods

### 3.1. Literature Search Strategy

**Figure 1:**
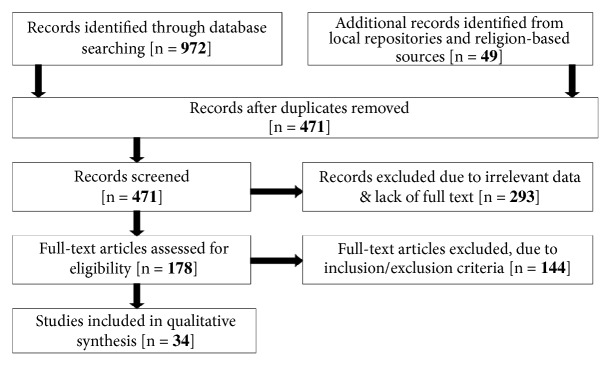
Flow of information retrieving strategy during identification, screening, and scrutinizing eligibility of the documents for the systematic review according to PRISMA suggestions.

Ethnobotanical studies from Ethiopia were systematically reviewed. Information included in this review spans from mid-1970s to 2017, which was the period when valid documentation on the Ethiopian TMs was undertaken. Knowledge of CAM for the treatment of various forms of cancer/malignancies in Ethiopia was retrieved from PubMed database (https://www.ncbi.nlm.nih.gov/pubmed-accessed on 25.09.2017) following “Preferred Reporting Items for Systematic Reviews and Meta-Analyses (PRISMA)” guidelines [100]. Electronic repository of the Addis Ababa University (AAU) (http://etd.aau.edu.et) and sources that make references to the TM knowledge of church-based religious teachers (‘debteras') were examined as additional sources (Figure 1). In the search process, keywords including, “ethnobiology”, “ethnobotany”, “ethnobotanical” “ethnomedicine”, “ethnomedicinal”, “medicinal plants”, “traditional healer”, “traditional medicine practitioner”, “traditional medicine”, “traditional herbal medicine “herbal medicine”, “food supplements” and “medico-cultural”, all with the term “Ethiopia” were used. After completing identification, screening and checking the eligibility of the literature for the systematic review, only those reporting forms of malignancies/cancer, neoplasms,* ‘*nekersa', tumor and swelling due to* ‘*nekersa' were analyzed. Throughout the review paper the term “cancer” or ‘nekersa' or “malignancy” is used to define a condition where cells of neoplastic features divide without control and can invade nearby tissues.

### 3.2. Inclusion and Exclusion Criteria

Information obtained from the database and other sources were scrutinized based on the following conditions. Published articles and unpublished thesis/dissertation on ethnomedicinal/ethnobotanical surveys reporting MPs used in Ethiopia to treat various forms of malignancies/cancer were included.

Researches reporting (i) CAM used for the treatment of single etiology; (ii) ethnoveterinary research reports; (iii) review articles; (iv) published in languages other than English; (v) lacking clear objectives and methodologies; (vi) abstract only or without full text access; (vii) experimental studies; (viii) articles lacking scientific plant names; (ix) research reports without voucher numbers of the specimens; and (x) ethnobotanical information with no reports of MP use for the treatment of malignancies/cancer and/or any neoplasm were excluded.

### 3.3. Data Extraction and Review Process

After the retrieval from the electronic databases, the research articles were imported to the ENDNOTE software version X7 (Thomson Reuters, USA) and the duplicates were removed. After the removal of the duplicates, all the imported articles and the additional files were checked against the inclusion/exclusion criteria. Following the confirmation of the eligibility for the systematic review, each document was carefully examined at a time. We extracted the following data from each eligible document: (i) taxonomic diversity (the scientific name of the species and family name), (ii) vernacular/local name of the plant, (iii) habitat of the plant, (iv) the growth form (habit of the MP), (v) parts of the MPs used for the remedy preparation and routes of administration, (vi) the forms of malignancies treated, and (vii) reports on the adverse effect(s); contraindications and antidotes were carefully extracted and analyzed. The citation frequency and mapping of their geospatial distribution were done. Data on the distribution of MPs in the Ethiopian flora region and the altitudinal range the plants grow was generated. Data extraction was carried out twice independently and the data sheet was further checked for methodological conformity and correction of any discordance. Descriptive statistics was used to summarize the findings.

## 4. Results

### 4.1. Taxonomic Diversity and Growth form and Distribution of the Anticancer MPs of Ethiopia

One hundred nineteen (119) species of anticancer MPs distributed in 98 genera and 57 families were retrieved from thirty-four (34) reports that fulfilled the inclusion/exclusion criteria (Table 1). Twenty-five (25) families were represented by two or more anticancer MPs, whereas 32 families were represented by a single species each (Appendix). The families Fabaceae (10.92 %) and Euphorbiaceae (10.08 %) were represented by the highest number of anticancer MP species followed by the families Asteraceae and Lamiaceae (5.04 % each). Eight (8) anticancer MP species were found to be widely used in different regions of the country, where the species* Plumbago zeylanica *(family Plumbaginaceae) being the most frequently used in areas including Jeldesa Cluster (Dire Dawa city administration), Mecha and Ghimbi Districts of Oromia Regional State and Zegie Peninsula, Amhara regional state (Table 2). The life forms of the anticancer MPs are constituted shrubs (42.02 %), herbs (35.29 %), trees (18.49 %), and climber/liana (4.2 %). A total of 89.08 % of the MPs are found in the wild habitats; home gardens make up 9.24 % and the remaining 1.68 % are found, both in the wild and home gardens. About 8.4 % of anticancer MP species are endemic, 86.56 % are indigenous, and 5.04 % are introduced from elsewhere.* Bidens macroptera (Sch Bip.) ex Chiov. Mesfin, Erythrina brucei *Schweinf.,* Euphorbia heterochroma *Pax.,* Galium boreo-aethiopicum *Puff.,* Lobelia rhynchopetalum *(Hochst.) Hemsl,* Millettia ferruginea *(Hochst.) Baker,* Pittosporum abyssinicum* Del.,* Plectocephalus varians (A. Rich) Jeffrey ex Cufod., Sideroxylon oxyacanthum *Baill, and* Vernonia leopoldi* (Sch. Bip. ex Walp.) Vatke are endemic to Ethiopia [39, 101, 102].

### 4.2. Geospatial Distribution of the Anticancer MPs

**Figure 2:**
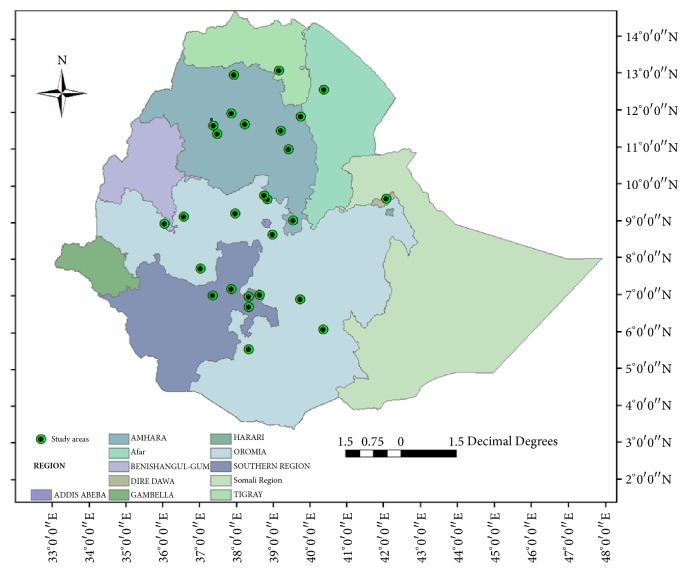
The geographical distribution of the Ethiopian anticancer traditional MPs.

According to the present systematic review, the eligible studies on anticancer MPs were reported mainly from the Oromia Regional State (37.04 %), Amhara Regional State (33.33 %), and Southern Nations, Nationalities and Peoples Regional (SNNPR) State (18.52 %) (Table 3). The frequency of reports across the regions and distribution in the Ethiopian Flora Region are shown in Figure 2 and Appendix.

### 4.3. Forms of Malignancies Most Frequently Treated by Traditional MPs

**Figure 3:**
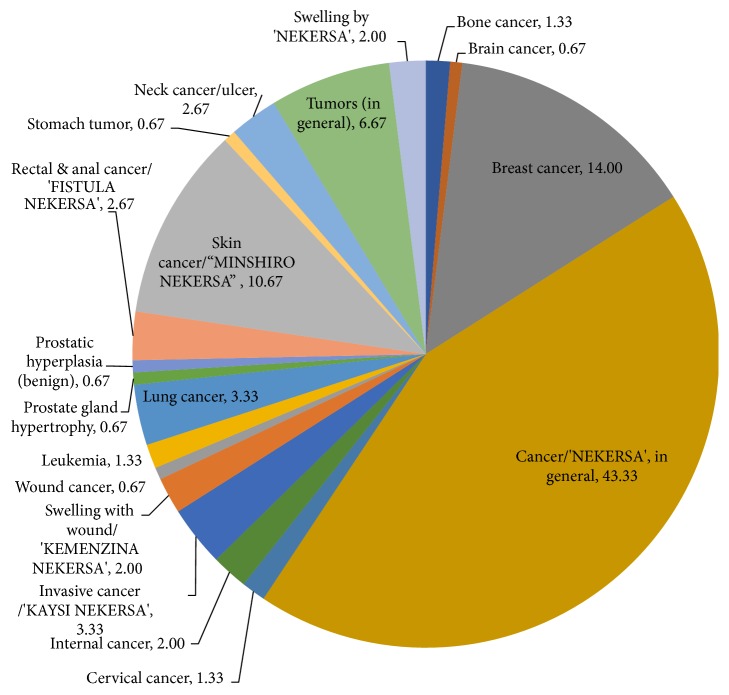
Types and frequency of cancer forms treated by traditional MPs in Ethiopia (%).

A variety of malignancies or cancer forms were reported to be treated with traditional MPs in Ethiopia. In the higher number of the reports (43.33 %), it was reported as “cancer*/ ‘*nekersa'” nonspecifically. Specific forms of cancer reported include breast cancer (14.0 %) and skin cancer/‘Minshiro nekersa' (10.67 %). The remaining list includes bone cancer, brain cancer, cervical cancer, rectal cancer, anal cancer, invasive cancer/* ‘*kaysi nekersa', swelling with wound/‘kemenzina nekersa', leukemia, and lung cancer among others (Figure 3).

### 4.4. Parts of the MPs Used for the Remedy Preparation and Routes of Administration

**Figure 4:**
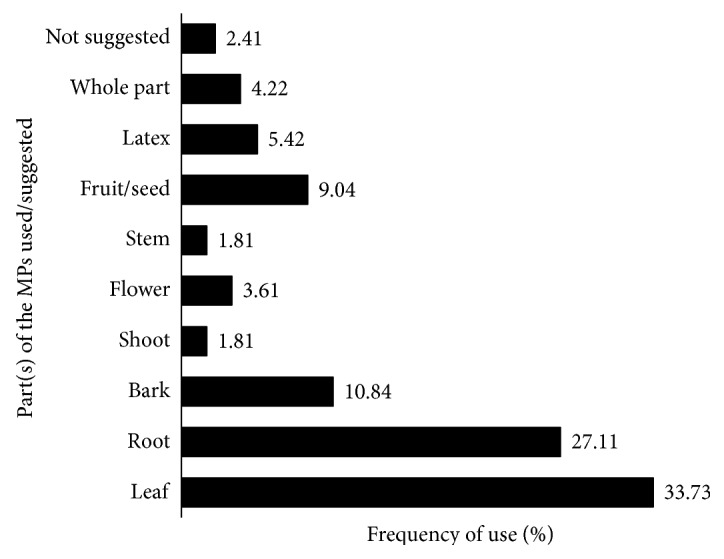
Part(s) of the anticancer MPs used/suggested for the treatment of various forms of cancer.

Analysis of the eligible ethnobotanical findings showed that various parts of the anticancer MPs were used for the remedy preparation. The most frequently harvested parts of the anticancer MPs are leaves (33.73 %) and roots (27.11 %). Debarking constituted 10.84 % whereas the whole parts of the MPs were used in as high as 4.22 % of the preparations. The stem and shoots were rarely collected (only 1.81 %, each) for the remedy preparation (Figure 4).

**Figure 5:**
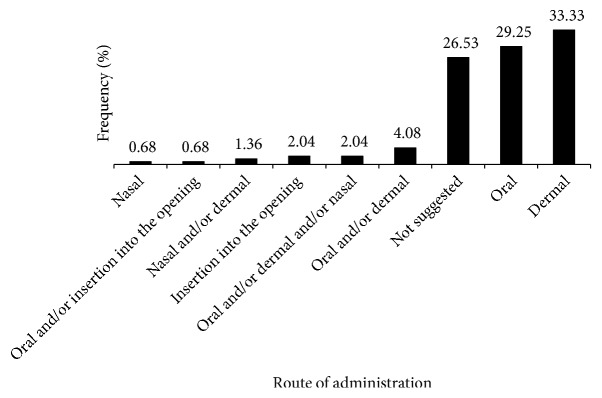
Routes of anticancer TM remedy administration.

From the various formulations and application procedures reported, the most preferred administration route of the TMs was dermal application (33.33 %) followed by oral (29.25 %). About 4.08 % of the applications recommend both oral and dermal routes of applications and in 26.53 % of the cases, the route of administration has not been specified (Figure 5). About 12.93 % of the preparations were made from dried plant materials whereas 87.07 % were prepared from fresh plant materials and water is the solvent that is mostly used to prepare anticancer TMs. About 4.76 % of preparations included honey as an additive to the remedy. Butter, flour of* ‘*teff', spicy stew, and sulfur (locally called* ‘*digne' in Amharic) among others were found to be mixed in about 4.08 % of the remedies. Common methods of dermal application included dressing the swelling with warm plant material, smearing on the wound, rubbing the affected area with the TM, tying the residue on the swelling, and giving a message with the fresh plant material preparation, as in the cases of breast and skin cancer.

### 4.5. Adverse Effects, Contraindications Implicated, and Antidotes

The present finding revealed that nine eligible studies discussed the existence and management approaches used by the TM practitioners in case the anticancer remedies cause any possible side effect(s). Besides, contraindications were also noted, pregnant women being the most commonly at risk. Out of 119 MP species compiled, eleven (11) species were found to have adverse side effects and/or contraindications. The most commonly implicated species was* Phytolacca dodecandra, *followed by* Croton macrostachyus. *Two studies have reported that any MP could result in mild adverse effects especially among the risk groups or in case of overdosing of the remedy. During such circumstances, drinking coffee, local beer and flax, and eating local food like* ‘SHIRO'* alleviates the symptoms [41, 69]. The findings are summarized along with the recommended antidote(s) (Table 4).

## 5. Discussion

These days, there is a higher level of dynamics in sociocultural transformations in Ethiopia. It is evident, however, that the communities by and large retained valuable knowledge of the MPs and their uses with regard to traditional healthcare. In the present investigation, 119 anticancer MPs were documented. This implies a wider usage of CAM among cancer patients in Ethiopia. This could be related to lack of full access to healthcare facilities. In addition, CAM usage is culturally accepted among Ethiopians as an effective cure and safe and is affordable. It is also perceived more efficacious against certain types of diseases including cancer [103–105]. However, the rapid population growth has resulted in an alarmingly high demand for agricultural land which in turn seriously threatens forest cover and hence the MPs. This makes designing and implementing of an insightful conservation program mandatory [29, 30, 106].

Consistent with various ethnobotanical studies, shrubs made up a larger proportion of the anticancer MPs (42.02 %) followed by herbs (35.29 %) [31, 44, 97, 107–109]. This may be explained by the fact that shrubs are perennial in the arid or subarid environments and may be available for use as MPs [64]. The dominance of certain life forms of MPs in different study areas might be explained by their availability and adaptation in the particular ecological setting and the dynamics involved due to bushy vegetation being left behind when the forest recedes.

Dominant families from which anticancer MPs are prepared were found to be Fabaceae (13 MP species, 10.92 %), Euphorbiaceae (12 MP species, 10.08 %), and Lamiaceae and Asteraceae (six MP species, 5.04 %, each). This could be attributed to the fact that these families are among the most widely distributed ones in the Ethiopian Flora Regions [31, 67, 110]. Since* Plumbago zeylanica *(Plumbaginaceae) is the anticancer MP species most widely used across the country, it may have a better healing potential over other anticancer MPs. This finding would make it a prime candidate for further in-depth experimental investigations.

The present review also revealed that roots (27.11 %), bark (10.84 %), and the whole part of the MPs (4.22 %) accounted for about 42.17 % usage in TM. This will significantly affect the sustainability of the MPs unlike use of aerial parts, such as leaves [91, 106, 111]. It was revealed that most remedies are prepared from fresh plant materials (87.07 %). This would result in the extensive exploitation of the MPs and in a long run and will compromise the sustainability of the MPs [55, 58, 111].

The finding that a significant proportion of the remedies were given orally (29.25 %) implies that the remedies are safe for systemic applications. However, in the present report over ten MP species used as anticancer agents across the country had contraindications and/or adverse effects calls for cautionary usage. In addition, there are experimental evidences on the toxicity of some anticancer MPs (e.g.,* Hagenia abyssinica* and the species from genus Vernonia) reported in the present study [112–114]. This indicates the need for acute and chronic toxicity investigations on the most commonly used traditional anticancer MPs.

The majority of anticancer MPs reported in the present study were from wild habitats (89.08 %). These habitats are vulnerable to demise by anthropogenic activities [57, 115]. As intermittent drought and wide spreading climatic changes are posing additional threats to the anticancer MPs of the country, they are limited to inaccessible areas. Therefore, as d'Avigdor and the coworkers suggested, the community must be encouraged to grow them in and around home gardens and backyard [46].

One of the greatest challenges in ethnomedicinal researches is the issue of access and benefit sharing (ABS) and that of intellectual property rights. WHO states that “agreements on the return of immediate and/or long-term benefits and compensation for the use of MP materials and associated community knowledge must be discussed and concluded” [116]. In the year 1986, there were over 6,000 TM practitioners in the country registered with the Ethiopian Ministry of Health [117]. However, there is no national guideline to address the regulatory situation [118]. The proclamation, which was issued based on the National Drug Policy in 1999 in Article 6, Sub-Article 8 states, “the Drug Administration and Control Authority shall prepare standards of safety, efficacy, and quality of TMs and shall evaluate laboratory and clinical studies in order to ensure that these standards are met”. It states that the authority shall also issue licenses for the use of TMs in the official health services. However, there were no official education and training systems to strengthen the TM practitioners in addition to the lack of regulation to address the issue of property rights and benefit sharing [117].

In Ethiopia, there have been attempts to implement ABS system of the Convention on Biological Diversity (CBD) according to the provisions in the Nagoya Protocol and other international agreements [119–121]. The country has acceded to it and developed a code of conduct to administer the ABS regime. However, there are several unmet issues requiring resolution [119, 120].

## 6. Conclusion

From the present study, it can be concluded that CAM practice is an integral part of the primary healthcare system of Ethiopians, where the traditional anticancer MPs are reported from different corners of the country. The majority of anticancer MPs are found in wild habitats. This shows that most MPs are vulnerable to destructive anthropogenic activities directed against forests and other environmental factors including climate variability. Thus, due attention should be given to conserving these valuable resources in addition to raising awareness of the community on how to use these plants sustainably.

We also call for the proper enforcement of the Nagoya Protocol with all its international recommendations and in accordance with the national setups in order to protect the TM knowledge and associated rights of indigenous communities. This will further not only grant an opportunity to salvage the indigenous knowledge held by the communities, but also help the scientific endeavor in plant-based anticancer and other diseases drug discoveries.

The efficacies of most Ethiopian traditional anticancer treatment claims with the MPs are not validated scientifically. Besides, only a small proportion of the MPs were reported to have side effects and/or contraindications. To avoid such overlooked health risks, we recommend further assessment on the safety of the anticancer MPs. Scientific investigation on the MP's potential toxicity and anticancer efficacy must also be made. This would possibly provide a lead material to a more thorough anticancer drug development researches.

## Figures and Tables

**Table 1:** The taxonomic distribution of anticancer MPs of Ethiopian flora.

**Family (N=57)**	**Genera N (**%**)**	**Species N (**%**)**
Fabaceae	9 (9.18)	13 (10.92)
Euphorbiaceae	7 (7.14)	12 (10.08)
Asteraceae	4 (4.08)	6 (5.04)
Lamiaceae	5 (5.10)	6 (5.04)
Ranunculaceae	3 (3.06)	5 (4.2)
Cucurbitaceae	3 (3.06)	3 (2.52)
Rosaceae	3 (3.06)	3(2.52)
Solanaceae	3 (3.06)	3(2.52)
Other 49 families	61 (62.24)	68(57.14)

**Total**	**98 (100**%**)**	**119 (100**%**)**

**Table 2:** Frequently cited anticancer MPs from different parts of Ethiopia.

**Scientific Name [Family]**	**Part(s) of the MP(s) used**	**Total reports**	**Areas/regions the MP(s) are used as anticancer agent**	**References**
*Plumbago zeylanica *[Plumbaginaceae]	Lf, Rt and Sh	5	Jeldesa Cluster (Dire Dawa) Mecha District, Zegie Peninsula, Ghimbi District and across the regions	[41–45]
*Brucea antidysenterica *[Simaroubaceae]	Lf, St and Br	3	Jimma Zone, Bale Zone and Fiche District	[46–48]
*Clematis hirsuta var. hirsute *[Ranunculaceae]	Lf, St and Br	3	Wondo Genet; Dega Damot and Blue Hora	[49–51]
*Croton macrostachyus *[Euphorbiaceae]	Lf	3	Wondo Genet, Hawassa city, and Dalle District	[37, 51, 52]
*Dodonaea angustirolia *[Sapindaceae]	Lf	3	Dalle District (different sites) and Debre Libanos monastery	[45, 52, 53]
*Dovyalis abyssinica *[Flacourtiaceae]	Fr, Br and WP	3	Fiche District, Dalle District and across the regions	[45, 54, 55]
*Vernonia leopoldi* (bitter leaf) [Asteraceae]	Lf	3	Jimma area, Bale Zone, and Zegie Peninsula	[44, 47, 48]
*Zanthoxylum chalybeum *[Rutaceae]	Lf and Rt	3	Hawassa city and Dalle District (different sites)	[52, 55, 56]

**Note:** Parts Used: Lf=Leaf; Rt=Root; Fr=Fruit; Br=Bark; Sh=Shoot; St=Stem.

**Table 3:** Geospatial distribution of the ethnobotanical studies that reported the use of CAM for the treatment of any form of malignancies/cancer atregional/city administration level.

**Region/city administration of the Federal Democratic Republic of Ethiopia **	**Frequency (**%**)**
Oromia Regional State	10 (37.04)
Amhara Regional State	9 (33.33)
SNNPR State	5 (18.52)
Tigray Regional State	1 (3.70)
Dire Dawa City Administration	1 (3.70)
Afar Regional State	1 (3.70)

**Total reports**	**27 (100)**

**Table 4:** Adverse/side effects, contraindication implicated, and possible antidote.

**Family**	**MP (s) involved**	**Contraindication (if any)**	**Adverse effect(s); [possible antidote recommended] (if any)**	**Reference**
Asteraceae	*Vernonia Amygdalina*,	Pregnant women	A headache and diarrhea	[50, 57]
Cucurbitaceae	*Zehneria scabra*	-	A headache, vomiting, and diarrhea [Taking shower]	[50, 57]
Euphorbiaceae	*Croton macrostachyus*	Pregnant women	A headache, vomiting, diarrhea, urination; [“Teff injera*”* and porridge are anecdotes]	[50, 57]
		-	Any; [milk whey as an antidote]	[58]
		Pregnant women	Nausea, vomiting and diarrhea	[59]
Fabaceae	*Calpurnia aurea*	-	headache	[57]
	*Millettia ferruginea*	Pregnant women	Nausea, vomiting and diarrhea	[59]
Lobeliaceae	*Lobelia rhynchopetalum*	-	Vomiting and diarrhea; [*“SHIRO WOT”* (sauce made of pulse grains) and powder of *Linum usitatissimum* infusion in water]	[60]
Menispermaceae	*Stephania abyssinica*	-	Any; [milk whey as an antidote]	[58]
Phytolaccaceae	*Phytolacca dodecandra*	Pregnant women	Overdosage would result in death	[53]
		Women at a child-bearing age	Could result in sterility in women	[46]
		-	Vomiting and diarrhea; [*“SHIRO WOT”* (sauce made of pulse grains) and powder of *Linum usitatissimum* infusion in water]	[60]
		Children and pregnant women	Vomiting and diarrhea; [milk and red “*TEFF” *porridge]	[50, 57]
		Pregnant women	Nausea, vomiting and diarrhea	[59]
Polygonaceae	*Rumex nervosus*	Children	Vomiting and diarrhea; [red “Teff' porridge]	[50, 57]
Ranunculaceae	*Clematis hirsuta var. hirsuta*	-	A headache, Sweating, and diarrhea	[50, 57]
Rosaceae	*Hagenia abyssinica*	-	A headache, vomiting, and diarrhea	[50, 57]
			[*“SHIRO WOT”* (sauce made of pulse grains) and powder of *Linum usitatissimum* infusion in water]	[60]

**Table 5:** Families of anticancer MPs with their scientific and local names, habitats, growth forms, parts used, application route and procedures, area of reporting, and forms of cancer treated and distribution in the Ethiopian Flora Region.

**Family**	**Scientific name of the plant **	**Vernacular name** ^**¥**^	**Habitat**	**G** **F** ^Ψ^	**PU** ^**§**^	**AR** ^**‡**^	**Application procedure**	**Cancer form described**	**The study area reported [Reference]**	**Distribution in the flora region (altitudinal range (m)) [Reference]**
Acanthaceae	*Barleria eranthemoides *R. Br. ex C.B. Clarke	Bilinjii (Oro)	W	Sb	Rt	Or	Dried or fresh root powder with water is given orally	Tumor	Fiche District, Oromia [54]	EW, AF, TU, GD, GJ, WU, SU, KF, GG, SD, BA, HA (500-1900) [61]
	*Hygrophila schulli* (Hamilt.) MR. & S.M Almeida	Bala noranti (Oro)	W	H	Rt	NA	NA	*‘*Nekersa*'*	Across the regions of Ethiopia [45]	EW, TU, GD, GJ, WU, SU, WG, IL, KF, GG, SD (550-2600) [61]
Aloaceae	*Aloe pirottae* Berger* (aloe) *	Iret (Amh)	W	Sb	Lf	Or	One tablespoon of pulp (leaf) is mixed with honey and eaten twice a day	Cancer	Sidama Zone, SNNP Region [62]	GG, SD, BA, HA (1300-1820) [63]
Amaranthaceae	*Celosia polystachia* (Forssk.) C.C. Towns.	NA	W	H	Lf	Or, Ns & De	The leaf of the plant is applied orally, nasally or topically	Breast cancer	Yalo District, Zone 4, Afar Region [64]	EW, WU, SU, KF, GG, SD, BA, HA, (300-1300) [65]
Amaryllidaceae	*Scadoxus multiflorus *(Martyn) Raf.	Dem astefit (Amh)	W	H	Rt	De	Used in combination with other herbs and applied topically	Internal cancer	Across the regions of Ethiopia [45]	EW, GD, SU, AR, WG, KF, IL, GG, SD, BA, HA (1000-3000) [63]
Anacardiaceae	*Lannea sp. *	Duduna (Age)	W	T	Br	De	Tied on the affected part	*‘*Kemenzina nekersa*'*/ swelling with wound	Across the regions of Ethiopia [45]	EW, TU, GD, GJ, WU, SU, AR, WG, KF, GG, SD, BA, ?HA (300-2200) [66]
Anthericaceae	*Anthericum angustifolium *Hochst. ex A.Rich	Wotet Ashir (Age)	W	H	Rt	De	Creamed on the surface	*‘*Minshiro nekersa*'*/ skin cancer	Across the regions of Ethiopia [45]	EW, TU, GD, WU (1300-3000) [63]
	*Chlorophytum tetraphyllum *(L.f) Baker	Ye-Eregna kolo (Amh)	W	H	Fr	Or	The fruit is pounded, cold macerated and drunk	*‘*Nekersa*'*	Across the regions of Ethiopia [45]	EW, TU, GD, GJ, WU, SU, WG, SD, BA (1250-3400) [63]
Apiaceae	*Ferula communis L. *	Doge (Amh) Etse-Tekhino (Ge'ez)	W	H	Rt	Or	The root is crushed and drunk with water	Lung cancer	Libo Kemkem District, Amhara Region [67]	EW, TU, GD, GJ, WU, SU, SD, BA, HA (1400-3250) [68]
					Rt	NA	NA	*‘*Nekersa*'*	Across the regions of Ethiopia [45]	
	*Foeniculum vulgare *Mill.	Ensilal (Amh)	HG	H	Rt	Or	Used in combination with other herbs and taken orally	Lung cancer	Across the regions of Ethiopia [45]	EW, TU, GD, GJ, WU, SU, AR, WG, KF, GG, SD, BA, HA (1600-2350) [68]
Apocynaceae	*Carissa spinarum *L.	Agam (Amh) Otila** (**Sid)	W	Sb	Fr	NA	NA	Cancer	Dale District, Sidama Zone, SNNP Region [52]	AF, EW, TU, GD, GJ, WU, SU, AR, WG,.KF, GG, SD, BA, HA (550-2500) [68]
	*Vinca major* L.	NA	HG	Sb	WP	NA	The whole areal part is used	Cancer	Loma and Gena bosa Districts Dawro zone, SNNP Region [59]	EW, SU (NA) [68]
Asclepiadaceae	*Huernia macrocarpa *(A.Rich) Sprenger	Yemidir kulkual (Amh)	W	H	Lx	In	The latex is mixed with ‘sumanfar*'* and inserted in to the cancer wound	Skin cancer	Gubalafto District, North Wello Zone, Amhara Region [69]	EW, TU, WU (1600-2400) [68]
	*Kanahia Laniflora *(Forssk.) R. Br.	Arust /tifirinde (Amh)	W	Sb	Lf & Lx	Or & De	Fresh leaf juice with latex is given orally or applied topically	Tumor	Mecha District, West Gojjam Zone, Amhara Region [42]	AF, EW, TU, GD, GJ, SU, AR, IL, KF, GG, SD, BA, HA, (600-2500) [68]
					WP	De	NA	*‘*Nekersa*'*	Across the regions of Ethiopia [45]	
Asparagaceae	*Asparagus africanus *Lam.	Yeset kest (Amh) Serati (Kam)	W	Sb	Rt	Or	NA	Breast pain/*'*nekersa'	Kembatta Tembaro (KT) Zone, SNNP Region [58]	EW, TU, GD, GJ, WU, SU, AR, WG, KF, GG, SD, BA, HA (700-3800) [63]
Aspleniaceae	*Asplenium trichomanes *L.	Etse-Anbessa (Ge'ez)	W	Sb	Rt	Or	NA	*‘*Nekersa*'*	Across the regions of Ethiopia [45]	BA, SU and possibly other regions (2900) [70]
Asteraceae	*Artemisia absinthium* L.	Ariti (Amh)	HG	H	Lf	Or	Mixed with Tenadam (*Ruta chalepensis*), and Zingibil (*Zingiber officinale*) made into an infusion, filtered and drunk	Cancer	Fiche District, North Shewa Zone, Oromia Region [46]	EW, TU, GD, SU, WG, HA (1700-2440) [71]
	*Bidens macroptera *(Sch Bip.) ex Chiov. Mesfin	Adey Abeba (Amh)	W	H	Fl	Or	Powdered flower part is used	Brain cancer	Libo Kemkem District, Amhara Region [67]	EW, TU, GD, GJ, WU, SU, AR, IL, KF, GG, BA, HA (1750-3600) [71]
	*Plectocephalus varians *(A. Rich) Jeffrey ex Cufod.	Este-Yohannes (Amh)	W	H	WP	NA	Whole fresh plant is squeezed and applied	Tumor	Mecha District, West Gojjam Zone, Amhara Region [42]	EW, TU, GD, GJ, SU, AR, WG, KF, GG, SD, BA, HA (1900-3600) [71]
	*Vernonia amygdalina *Del.	Grawa (Amh) Hecho (Sid)	W	Sb	Sh	Or	Tender shoots are pounded and the juice squeezed from the pulp is drunk	Cancer (as chemoprevention)	Sidama Zone, SNNP Region [62]	EW TU, WU, GD GJ, SU, WG, IL, KF, GG, SD, BA, HA (650-3000) [71]
	*Vernonia hymenolepis *A. Rich.	Qilxuu (Oro)	W	Sb	Lf	NA	NA	Tumor	Jimma Zone and Bale Zone, Oromia Region [47, 48]	GJ, SU, AR, WG, IL, KF, GG, SD, BA, HA (1200-3000) [71]
	*Vernonia leopoldi* (Sch. Bip. ex Walp.) Vatke (bitter leaf)	Merara kitel (Amh)	W	Sb	Lf	NA	NA	Tumor	Jimma Zone and Bale Zone, Oromia; Zegie Peninsula, Northwestern Ethiopia, Amhara Region [44, 47, 48]	TU, GD, GJ, WU, SU, WG, KF, HA, GG (1850-2850) [71]
Balanitaceae	*Balanites aegyptiaca* (L.) Del.	NA	W	T	Lf	Or, Ns & De	NA	Breast cancer	Yalo District, Zone 4, Afar Region [64]	EW, TU, WU, SU, AR, HA, IL, GG, SD (700-1800) [66]
	*Balanites rotundifolia* (van Tieghem) Blatter	NA	W	Sb	Lf	OR & De	NA	Breast cancer	Yalo District, Zone 4, Afar Region [64]	AF, GG, SD (300-1500) [66]
Berberidaceae	*Berberis holstii *Engl. GB	Yeset af / Zinkila (Amh)	W	Sb	Br	De	Bark of the root; used in combination with other herbs and applied topically	*‘*Fistula nekersa'	Across the regions of Ethiopia [45]	EW, TU, WU, SU (2406-3200) [65]
Boraginaceae	*Cordia africana *Lam.	Wanza (Amh)	W/HG	T	Br	De	The bark of *Cordia africana* with the root of *Plumbago zeylanica *is powdered, mixed with butter and creamed on the affected part until recovery	Tumors (‘nekerse')	Debark District, North Gondar Zone, Amhara Region [72]	EW, TU, GD, GJ, WU, SU, AR, WG, IL, KF, GG, SD, BA, HA (700-2550) [61]
					Br	NA	NA	Cancer	Dale District, Sidama Zone, SNNP Region [52]	
	*Ehretia cymosa *Thonn.	Mukerba (Amh)	W/HG	Sb	Br		Bark of the root is used in combination with other herbs and applied topically	*‘*Fistula nekersa'/ rectal & anal cancer	Across the regions of Ethiopia [45]	EW, *TU, *GJ, WU, SU, AR, WG, IL, KF, GG, SD, BA, HA (900-2350) [61]
Brassicaceae	*Brassica carinata *A. Br.	Gomen zer (Amh)	HG	H	Sd	De	The seed of *B. carinata* with seed and leaf of *Tarenna graveolens* (galo – Amh) is crushed, powdered, mixed with honey and creamed on the affected part	Skin cancer	Debark District, North Gondar Zone, Amhara Region [72]	EW, GD, SU, IL, HA (1350-3850) [65]
Capparidaceae	*Cadaba farinosa* Forssk.	Qalaanqaal (Som)	W	Sb	Lf	Or	NA	Breast cancer	Yalo District, Zone 4, Afar Region[64]	AF, EW, TU, GD, GJ, WU, SU, AR, WG, IL, KF, GG, SD, BA (sea level to 2000) [65]
Caryophyllaceae	*Silene macrosolen* A. Rich	NA	W	H	Lf	Or	NA	Breast cancer	Yalo District, Zone 4, Afar Region [64]	TU, GD, WU, SU, AR SD BA, HA (1900-3600 ) [65]
Celastraceae	*Maytenus senegalensis *(Lam.) Exell	Atat (Amh)	W	Sb	Lf	De	It is pasted on the affected area	Cancer	Gondar Zuria District, Amhara Region [73]	EW, TU, WU, GD, GJ, WG, SU, HA, AR, BA, IL, KF, GG, SD (380-2440) [66]
Combretaceae	*Combretum Collinum *Fresen.	Abalo (Amh)	W	T	Lf	De	The leaves of *Combretum Collinum* are pounded, powdered and applied on wound or tumor	Wound and Tumors (‘nekersa*'*)	Debark District, North Gondar Zone, Amhara Region [72]	GJ, SU, (Gibe Gorge),WG, IL, KF, GG, SD, BA, HA (450-1950) [74]
Cucurbitaceae	*Lagenaria siceraria *(Molina) Standl	Basu baaqula (Sid)	HG	H	Lf	De	The leaves are crushed, squeezed, and applied on the wound	‘Nekersa*'*	Dega Damot District, Amhara Region [50]	GD, GJ, TU, SU, AR, IL, KF, GG, BA, HA (300-2800) [74]
					Rt	Or	Dry root is pounded, powdered and drunk orally	Cancer	Hawassa city, Sidama Zone, SNNP Region [56]	
	*Momordica friesiorum* (Harms) C. Jeffrey	Wof tech (Amh)	W	H	Rt	De	Used in combination with other herbs and applied topically	*‘*Kaysi nekersa*'*/ invasive cancer	Across the regions of Ethiopia [45]	SU, AR, GG, BA, HA (1180-2800) [74]
	*Zehneria scabra *(L.F. Sond)	Areg resa (Amh) Etse-Sabek (Ge'ez)	W	H	Lf & Rt	NA	NA	‘Nekersa*'*	Across the regions of Ethiopia [45]	EW, TU, GD, GJ, WU, SU, AR, WG, IL KF GG, SD, BA, HA (1200-3580) [74]
Euphorbiaceae	*Acalypha fruticosa* Forssk	NA	W	Sb	Lf	Or, Ns & De	Crushed and applied oral, nasal or topical	Breast cancer	Yalo District, Zone 4, Afar Region [64]	EW, WU, SU, GG, SD, HA (435-1800) [74]
	*Bridelia micrantha *(Hochst.) Baill.	Yenebir tifir (Amh)	W	Sb	Rt	NA	NA	‘Nekersa'	Across the regions of Ethiopia [45]	GD, GJ, SU, AR, WG, IL, KF, GG, SD, BA (1050-2200) [74]
	*Clutia abyssinica *Jaub. and Spach.	Fiyele-fej (Amh) Este-mefrih (Ge'ez)	W	H	WP	De	The whole part often together with *C. richardiana *and* C. robusta; *used topically	‘Nekersa*'*	Across the regions of Ethiopia [45]	TU, GD, WU, GJ, SU, AR, KF, SD, BA, HA (1450-2950) [74]
	*Croton macrostachyus *Del.	Bisana (Amh) Masina (Sid)	W	Sb	Lf	De	Dry/fresh leaves are pounded, powdered and put on the affected part	Wound cancer	Wondo Genet District, Sidama Zone, SNNP Region [51]	
					Lf	In	Fresh leaves are crushed and inserted into the wound	Skin cancer	Hawassa city, Sidama Zone, SNNP Region [37]	EW, TU, GD, GJ, WU, SU, AR, WG, IL, KF, SD BA HA (500-2350) [74]
					Lf	NA	NA	Cancer	Dale District, Sidama Zone, SNNP Region [52]	
	*Euphorbia ampliphylla *Pax.	Qulquale (Amh)	W	T	Fl	De	The flower of *Euphorbia ampliphylla* is pounded, powdered and mixed with honey and creamed on the affected part	Skin cancer (‘lemtse*'*)	Debark District, North Gondar Zone, Amhara Region [72]	TU, GD, GJ, WU, SU, IL, KF, SD, HA (1200-2700) [74]
					Rt & Lx	NA	Root and latex of *Euphorbia abyssinica, *together with* Euphorbia caandelabrum & Kotschy and Euphorbit oboalifolia *	‘Nekersa*'*	Across the regions of Ethiopia [45]	
	*Euphorbia dalettiensis* M *Gilbert*.	Kelekol (Amh)	W	Sb	Rt & Lx	NA	NA	Invasive ‘nekersa*'*	Across the regions of Ethiopia [45]	HA (1200) [74]
	*Euphorbia lathyris *L.	Hadaamii (Oro)	HG	H	St	De	Stem of *Euphorbia lathris *is chopped and fumigated to ulcerated breast due to cancer	Breast ulcer	*Chelya District, West Shewa,* Oromia Region [75]	SU, HA (*c *2000) [74]
	*Euphorbia platyphyllos *L.	Anitrfa (Amh)	W	H	Lx	De	Fresh latex is applied on the tumor topically	Tumor	Mecha District, West Gojjam Zone, Amhara Region [42]	GJ, SU, AR (2000-3200) [74]
	*Euphorbia polyacantha* Boiss.	Carricho (Sid)	W	Sb	Lx	De	Its latex is squeezed and creamed on the affected area	Skin cancer (tumor)	Delanta District, Northwestern Wello, Amhara Region [60]	EE, EW, TU, WU, SU, BA, HA (1200-2250) [74]
	*Euphorbia tirucalli *L.	Kinchib (Amh) Shuraamo carre (Sid)	W	T	Lx	De	Latex dropped on the affected part	Skin cancer	Wondo Genet District, Sidama Zone, SNNP Region [51]	EW, TU, WU, SU, IL, GG, SD, BA, HA (1300-2000) [74]
					NA	NA	NA	Unidentified swellings/ Neck Cancer	Debre Libanos monastery, North Shewa *Zone*, Oromia Region [53]	
	*Phyllanthus ovalifolius Forssk.*	Sosiye (Amh)	W	Sb	Rt	NA	NA	‘Nekersa*'*	Across the regions of Ethiopia [45]	GD, GJ, WU, WG SU, AR, IL, KF, GG, SD, BA, HA (900-2750) [74]
	*Ricinus communis *L.	Gulo (Amh) Qonbo”o (Sid)	HG	H	Rt	Or	Fresh root is chewed and swallowed	Breast cancer	Hawassa city, Sidama Zone, SNNP Region [55, 56]	TU, GD, WU, SU, WG, IL, KF, GG, SD, BA, HA (400- 2500) [74]
					Lx	NA	NA	‘Nekersa'	Across the regions of Ethiopia [45]	
Fabaceae	*Acacia oerfota* (Forssk.) Schweinf.	Seraw (Tig)	W	Sb	Lf	Ns & De	Crushed and applied nasally and/or topically	Breast cancer	Yalo District, Zone 4, Afar Region [64]	AF, EW, TU, WU, SU, BA, HA, SD (100-1600) [66]
	*Acacia tortilis* (Forssk.) Hayne.	Seraw (Tig)	W	T	Lf	Ns & De	Crushed and applied nasally and/or topically	Breast cancer	Yalo District, Zone 4, Afar Region [64]	AF, EW, TU, WU, SU, AR, HA, BA (600-1900) [66]
	*Calpurnia aurea *(Alt.) Benth.	Digita (Amh)	W	Sb	Lf	Or	The powdered leaf of the plant is mixed with root of* Cucumis ficifolius *(Yemidir Embuay) is cold macerated and given orally	Unidentified swellings/ Cancer	Debre Libanos monastery, North Shewa *Zone*, Oromia Region [53]	EW, TU, GD, WU, GJ, WG, SU, AR, BA, HA, KF, GG, SD (1650-3000) [66]
	*Colutea abyssinica *Kunth & Bouché	Duaduate (Amh)	W	Sb	Rt & Sd	De	Used in combination with other herbs and applied topically	Cervical and rectal cancer	Across the regions of Ethiopia [45]	EW, TU, GD, WU, SU, AR, BA, HA, SD (1600-4000) [66]
	*Dichrostachys cinerea* (L.) Wight et Am.	Ader (Amh)	W	Sb	Rt	Or	Root of the plant is crushed and taken orally	Skin bleaching (cancer)	Yalo District, Zone 4, Afar Region [64]	EW, TU, WU, GJ, WG, SU, AR, BA, HA, KF, GG, SD (450-2000) [66]
	*Erythrina brucei *Schweinf.	Kuara /Korch (Amh) Welekko (Sid)	W	T	Br	NA	NA	Cancer	Dale District, Sidama Zone, SNNP region [52]	WU, WG, GJ, SU, BA, HA, IL, KF, GD, GG, SD (1400-2600) [66]
	*Indigofera oblongifolia* Forsk.	NA	W	Sb	Lf	Or & De	Leaf of the plant is used orally and as a body wash	Breast cancer	Gubalafto District, North Wello Zone, Amhara Region [69]	AF, EW, HA, BA, KF, GG, SD (up to 1200) [66]
	*Millettia ferruginea *(Hochst.) Baker	Birbira (Amh) Hengedicho** (**Sid**)**	W	T	Br	Or	NA/ The bark is washed, pounded, filtered and given orally	Cancer	Dale District, Sidama Zone, SNNP region [52]	WG, SU, HA, BA, IL, KF, SD (1600- 2500) [66]
	*Senna alexandrina *Mill.	Mekerbaa (Oro)	W	Sb	Br	De	The bark is pounded and creamed on the swelling	Nekersa	Across the regions of Ethiopia [45]	AF, EW (0-1400) [66]
	*Senna italica* Mill.	NA	W	H	Lf	Or & De	Leaf of the plant is used orally and as a body wash	Breast cancer	Yalo District, Zone 4, Afar Region [64]	AF, EW, TU, GD (0-1850) [66]
	*Senna septemtrionalis *(Viv.) Irwin & Barneby	NA	W	H	Lf	Or	The Leaf is crushed in water and the filtrate is drunk	Lung cancer	Gubalafto District, North Wello Zone, Amhara Region [69]	SU, AR, HA, IL, KF, SD (1700-2400) [66]
	*Senna singueana* (Del.) Lock	Key enchet (Amh) Busha (Age)	W	Sb	Lf & Br	De	Applied topically	‘Minshiro nekersa'	Across the regions of Ethiopia [45]	EW, TU, GD, WU, GJ, SU, SD (1500-2400) [66]
	*Sesbania sesban *L.Merr.	Bofefe – Amh	W	Sb	Rt	NA	NA	‘Nekersa*'*	Across the regions of Ethiopia [45]	AF, EW, TU, GD (Sudan border), WU, GJ, WG, SU, AR, HA, IL, KF, GG, SD (300-2000) [66]
Flacourtiaceae	*Dovyalis abyssinic a *(A. Rich.) Warb	Koshim (Amh)	W	Sb	Fr	Or	Six to ten fruits are eaten	Cancer	Fiche District, Oromia [54]	
					Br	Or	The raw bark of *Dovyalis abyssinica* is chewed and swallowed	‘Muje*'* /Lymphatic tumor	Dale District, Sidama Zone, SNNP Region [55]	TU, GD, GJ, WU, SU, AR, GG, SD, BA, HA (1700-3000) [65]
					WP	De	Creamed on the affected part	‘Nekersa*'*	Across the regions of Ethiopia [45]	
Iridaceae	*Gladiolus candidus *(Rendle), Goldblatt	Milas Golgul (Amh) Hanxxaye (Oro)	W	H	Rt	Or & De	The root is powdered and applied on the wound, or the powder is mixed with water and drunk	‘Nekersa*' *(cancer)	Across the regions of Ethiopia; Dega Damot District, Amhara Region [45, 50]	AR, SD, BA, HA (1450-2250) [63]
Juncaceae	*Juncus effusus *L.	Etse felatsut (Amh)	W	H	Rt	De	Used in combination with other herbs and applied topically	*‘*Kaysi nekersa*'*	Across the regions of Ethiopia [45]	AR, BA (2400-3120) [63]
Lamiaceae	*Ajuga integrifolia *Buch.-Ham. ex D. Don	Etse-libawit (Ge'ez) Harma guusaa (Oro)	W	Hb	Lf	De	Applied on affected breast	Breast cancer massage	Jimma Zone, Oromia Region [47]	EW, TU, GD, GJ, WU, SU, KF, SD, BA, HA (1500-3400) [61]
	*Clerodendrum myricoides *(Hochst.) Vatke	Misrichi (Amh) Mardhisiisaa (Oro) Ma'niisa (Sid)	W	H	Lf	Or	The leaf part will be pounded, mixed with honey and drunk; or its root boiled often mixed with the shoot of Z*anthoxylum chalybeum *	Cancer (Leukemia)	Dale District, Sidama Zone, SNNP Region [55]	TU, GD, WU, SU, AR, WG, IL, KF, GG, SD, HA (700- 2600) [61]
	*Leonotis ocymifolia *(Burm.f.) Iwarsson	Ye feres zeng (Amh)	W	Sb	Lf	De	Chopped leaves are applied to the ulcer for 24 hours	Ulcer of the neck (‘nekersa*'*)	Fiche District, North Shewa Zone, Oromia Region [46]	EW, TU, GD, WU, GJ, SU, AR, WG, IL, KF, GG, SD, SA, HA (500-3700) [61]
	*Leonotis raineriana* De FC (Vis.)	Ras kimir (Amh)	W	Sb	Rt	NA	Often used with *Leonotis Africana *and an application is not given	*‘*Minshiro nekersa*'*	Across the regions of Ethiopia [45]	EW, TU, GD, WU, GJ, SU, AR, WG, IL, KF, GG, SD, SA, HA (500-3700) [61]
	*Salvia nilotica* Jacq.	Hulegeb (Amh)	W	H	Rt	NA	NA	‘Nekersa*'*	Across the regions of Ethiopia [45]	All Flora region, except AF (1300-3800) [61]
	*Satureja abyssinica *(Benth.) Briq.	Este meaza (Amh)	W	Sb	Lf	De	Used in combination with other herbs and applied topically	Internal cancer	Across the regions of Ethiopia [45]	EW, TU, GD, GJ, WU, SU, AR, KF, GG, SD, BA, HA (900–2700) [61]
Linaceae	*Linum usitatissimum *L.	Telba (Amh)	HG	H	Sd	Or	NA	Breast pain/*'*nekersa*'*	Kembatta Tembaro (KT) Zone, SNNP Region [58]	Throughout the highlands (1600-3800) [65]
Lobeliaceae	*Lobelia giberroa* Hemsl.	Jibira (Amh)	W	T	Lx	NA	NA	‘Nekersa*'*	Across the regions of Ethiopia [45]	EW, TU, GD, SU, WG, IL, KF, GG, SD, HA, BA (1700-2800) [61]
	*Lobelia rhynchopetalum* Hemsl.	Etse-kemun (Ge'ez)	W	T	Rt	De	Used in combination with other herbs andapplied topically	*‘*Minshiro nekersa*'*	Across the regions of Ethiopia [45]	GD, GJ, SU, AR, BA, HA (3000-4350) [61]
Loganiaceae	*Buddleja polystachya* Fresen	Anfar (Amh)	W	Sb	Lf	Or	Pounded, cold macerated and taken orally	Cancer	Dale District, Sidama Zone, SNNP Region [55]	AF, TU, GD, GJ, WU, SU, AR, WG, KF, SD, BA, HA (700-3300) [68]
	.	Bullaancho (Sid)			Lf	NA	NA	*‘*Minshiro nekersa*'*	Across the regions of Ethiopia [45]	
Malvaceae	*Malva verticillata* L.	Lut (Amh)	W	H	Lf	De	The leaf is crushed, warmed on fire and tied on the swelling	Swelling by ‘nekersa*'*	Ada'a District, East Shewa Zone Oromia Region [31]	EW, TU, GD, GJ, WU, SU, AR WG, KF, SD, BA, HA (l600-4000) [74]
					NA	NA	NA	Unidentified swellings/ Neck Cancer	Debre Libanos monastery, North Shewa *Zone*, Oromia Region [53]	
	*Sida schimperiana *Hochst. ex. A. Rich.	Chefreg (Amh)	W	Sb	Lf & Rt	De	The leaf and root of *Sida schimperi* is pounded, powdered and then applied on affected part	Wound and Tumors (*‘*nekersa*'*)	Debark District, North Gondar Zone, Amhara Region [72]	EW, TU, GD, GJ, SU, WG, SD, BA HA (1500-2600) [74]
					Rt	Or	The juice of freshly squeezed root is mixed with honey	Breast cancer	Nekemte Town, East Wellega, Oromia Region [76]	
Meliaceae	*Ekebergia capensis *Sparm	Duduna (Amh) Simboo (Oro) Goddiicho (Sid)	W	Sb	Rt & Br	NA	NA	‘Nekersa*'*	Across the regions of Ethiopia [45]	TU, WU, GD, GJ, WG, SU, AR, IL, KF, SD, BA, HA (1680-3000) [66]
					Fr	Or	The fruit of the plant is pounded, filtered and drunk	Cancer	Dale District, Sidama Zone, SNNP Region [55]	
Melianthaceae	*Bersama abyssinica *Fresen.	Azamir (Amh) Xeweerrakko (Sid)	W	T	Br	Or	The bark of the plant pounded boiled and a small amount of the preparation is drunk	Cancer	Dale District, Sidama Zone, SNNP Region [55]	TU, GD, WU, WG, GJ, SU, IL, KF, AR, HA, BA, SD (1700-2715) [66]
Menispermaceae	*Stephania abyssinica *(Dillon & A. Rich.) Walp.	Engochit (Amh) Etse Eyesus (Ge'ez) Kalaala (Sid)	W	H	Lf	De	Fresh leaves are rubbed by hand and droplets are applied on the skin	Skin cancer	Wondo Genet District, Sidama Zone, SNNP Region [51]	EW, TU, GD, GJ, WU, SU, AR, WG, KF, IL, GG, SD, HA (1450-3400) [65]
Moraceae	*Dorstenia barnimiana *Schwienf.	Work Bemeda (Amh)	W	H	Rt	In	Small opening is made in the affected area and the root is inserted	Cancer	Zegie Peninsula, Northwestern Ethiopia, Amhara Region [44]	GD, SU, HA, SD, BA, IL, GG (500-2450) [66]
	*Ficus palmata* Forssk.	Beles (Amh)	W	Sb	Lf & Rt	De	Its root and leaves with bulb of *Allium sativium*, fruits of *Lagenaria siceraria* crushed together, backed with powder of teff and then applied on wounds	Skin cancer (‘lemtse*'*)	Minjar-Shenkora District, North Shewa Zone, Amhara Region [77]	EW, TU, GD, GJ, WU, SU, HA, AR, KF (1000-2400 ) [66]
					Br	NA	NA	*‘*Kaysi nekersa*'*	Across the regions of Ethiopia [45]	
	*Ficus sur *Forssk.	Shola (Amh)	W	T	Rt, Br & Fr	NA	NA	*‘*Kemenzina nekersa*'*	Across the regions of Ethiopia [45]	EW, TU,WU, GD, GJ, WG, SU, HA, AR, IL, KF, SD (1400-2500) [66]
Myrsinaceae	*Myrsine africana *L.	Quechemo (Amh)	W	Sb	Fr	Or	Dried fruit with dried leaf of *Osyris quadripartita*, powdered, mixed with little water is given orally	Cancer	Fiche District, North Shewa Zone, Oromia Region [54]	EW, TU, GD, GJ, WU, SU, AR, KF, SD, BA, HA (1900-3800) [68]
	*Myrsine Melanophloeos * (L.) R. Br.	Morocho (Sid)	W	T	Lf	Or	The leaf (often mixed with *Olea capensis*) is pounded, cold macerated and drunk	Cancer (leukemia)	Dale District, Sidama Zone, SNNP Region [55]	TU, GD, GJ, SU, AR, WG, GG, BA (2500-3750) [68]
Oleaceae	*Olea capensis *L.f.	Seettaame (Sid)	W	T	Sh	Or	The shoot part is boiled, mixed with honey and drunk; Shoot of *Zanthoxylum chalybeum and Clerodendrum myricoides* are often boiled together	Cancer	Dale District, Sidama Zone, SNNP Region [55]	GD, SU, AR, IL, KF, SD, BA (1350- 3200) [68]
	*Olea europaea* subsp. *Cuspidate *(Wall. ex. G. Don) Cif.	Woira (Amh)	W	T	Fr	Ns	The dried fruit and *Embelia schimperi *fruit powder with water is given nasally before food	Tumor	Mecha District, West Gojjam Zone, Amhara Region [42]	AF, EW, TU, GD, WU, SU, KF, GG, SD, BA, HA, (1250-3000) [68]
Phytolaccaceae	*Phytolacca dodecandra* L 'Herit.	Endod (Amh)	W	Sb	Lf & Rt	NA	Root and leaf of the plant is used by chopping and pounding;	Cancer/swelling of gland	Loma and Gena bosa Districts Dawro zone, SNNP region [59]	EW, TU, BA,GG, GD, WU, GJ, WG, SU, IL, KF, AR, SD, HA (1500-3000 ) [65]
Pittosporaceae	*Pittosporum abyssinicum *Del.	Lola (Amh) Boncho (Sid)	W	Sb	Br	NA	NA	Cancer	Dale District, Sidama Zone, SNNP region [52]	GD, SU, BA, HA (2300-3200) [66]
Plantaginaceae	*Plantago lanceolata *L.	Qorxobi (Oro)	W	H	Sd	De	Dried seeds are crushed, powdered and applied to the cancer wound	Cancer	Hawassa city, Sidama Zone, SNNP Region [56]	EW, TU,GD, GJ, WU, SU, WG, IL, KF, GG, SD, BA (1200-3200) [61]
Plumbaginaceae	*Plumbago zeylanica *L.	Amera (Amh) Aftuh (Tig) Mexres (Som)	W	H	Lf	Or	Leaf of *P. zeylanica *is squeezed and the juice is taken orally	Cancer	Ghimbi District, West Wollega Zone, Oromia Region [43]	
					Rt	De	Root powder mixed with “digne” (sulfur) is applied/ dressed with root paste	Cancer	Zegie Peninsula, Northwestern Ethiopia, Amhara Region [44]	
					Sh	Or	Fresh shoot boiled with water is given orally	Stomach tumor	Mecha District, West Gojjam Zone, Amhara Region [42]	AF, EW, TU, GD GJ, WU, SU, AR, IL, GG, SD, BA, HA (700-2200) [61]
					Rt	NA	NA	*‘*Kaysi nekersa*'*	Across the regions of Ethiopia [45]	
					Rt	Or & De	The root part is boiled and consumed orally and also applied topically	Bone cancer	Jeldesa Cluster, Dire Dawa city Administration [41]	
Podocarpaceae	*Afrocarpus falcatus *(Thunb.) C.N.Page	Zigba (Amh)	W	T	Lf	NA	NA	Cancer	Dale District, Sidama Zone, SNNP region [52]	TU, GD, GJ, WU, SU, AR, IL, KF, GG, WG, SD, BA, HA (1350-2900) [78]
Polygonaceae	*Oxygonum sinuatum* (Meisn.) Dammer	Kurnchit (Amh)	W	H	WP	De	Whole part is creamed on the affected part	*‘*Nekersa*'*	Across the regions of Ethiopia [45]	EW, TU, GD, GJ, SU, GG, SD, BA, HA (600-2500) [65]
	*Rumex abyssinicus *Jacq.	Mekmeko (Amh) Este-berhan (Ge'ez) Shiishoone (Sid)	HG	H	Rt	De	The root is pounded and creamed on the swelling	*‘*Nekersa*'*	Across the regions of Ethiopia [45]	EW, TU, GD, GJ, SU, AR, WG, KF, IL, GG, SD, BA, HA (1200-3300) [65]
					Rt	Or	Root powder is mixed in spicy stew to increase its power of curing and taken orally	Cancer	Seharti Samre District, Southern Tigray Region [57]	
	*Rumex nervosus *Vahl.	Huhot/Embuacho (Amh) Dhangaggoo (Oro)	W	Sb	Lf	De	Leaves are crushed and pasted on the affected area	Breast cancer	Seharti Samre District, Southern Tigray Region [57]	EW, TU, GD,, GJ, WU, SU, AR, GG, SD, HA (400-3300) [65]
Punicaceae	*Punica granatum *L.	Roman (Amh)	HG	Sb	Fr	Or	The fruit is crushed and eaten	Cancer	Libo Kemkem District, Amhara Region [67]	EW, SU, KF, and probably elsewhere (1000-2450) [65]
Ranunculaceae	*Clematis bracteata *(Roxb.) Kurz	Yeazo Areg (Amh)	W	Cl	WP	De	The plant is crushed and mixed with butter and applied	Cancer	Bale area, Southeast Ethiopia, Oromia Region [79]	GD, GD/GJ, GJ, SU, WG, KF, IL, SD (1350-3300 ) [65]
	*Clematis hirsuta var. hirsuta./*Perr. and Guill.	Nech yeazo Areg (Amh)	W	Cl	Lf, St & Br	Or	Leafs/stem and bark are mixed with leafs of *Plectranthus ingiarius* then pounded, filtered and drunk	Breast cancer	Wondo Genet District, Sidama Zone, SNNP Region [51]	
					Lf	De	The leaves are crushed and applied on the swelling as bandage	Swelling by *‘*Nekersa*'*	Dega Damot District, Amhara Region [50]	EW, TU, GD, GJ, WU, SU, AR, IL, KF, SD, BA, HA (850- 3200) [65]
					Lf	Or & In	The leaves are pounded to make a solution and half of small glass is drunk; certain amount of the solution is applied into the hole of the wound using syringe or other domestic material; the residue is put on the opening of the wound.	Bone cancer	Blue Hora District, Borana Zone, Oromia Region [49]	
	*Clematis simensis *Fresen.	Fiide (Sid)	W	Cl	Lf	De	The leaves are crushed, powdered and then creamed	Cancer	Libo Kemkem District, Amhara Region [67]	GD, TU, WU, GJ, SU, AR, WG, KF, GG, SD, BA, HA (1500-3350) [65]
					Lf	Or	Leaves of the plant will be macerated and drunk	Cancer	Dale District, Sidama Zone, SNNP Region [55]	
	*Ranunculus multifidus *Forssk.	Etse Siol (Amh/Ge'ez)	W	H	Lf	De	The leaf is powdered and dressed externally	*‘*Nekersa*'* (Cancer)	Debre Libanos monastery, North Shewa *Zone*, Oromia Region [53]	EW, TU, GD, GJ, WU, SU, AR, WG, IL, KF, SD, BA, HA (1200-3800)[65]
	*Thalictrum rhynchocarpum *Dill. & A. Rich.	Sire bizu (Amh)	W	H	Rt	De	Used in combination with other herbs and applied topically	‘Kemenzina 'nekersa'	Across the regions of Ethiopia [45]	TU, GD, GJ, SU, AR, WG, IL, KF, HA (1600-3050) [65]
Rhamnaceae	*Ziziphus mauritiana* Lam.	NA	W	Sb	Lf	Or	NA	Breast cancer	Yalo District, Zone 4, Afar Region [64]	IL, GG, SD, SD-BA, HA (400-1600) [66]
	*Ziziphus spina-christi *L. Desf.	Geba (Amh/Age) Qurqura (Oro)	W	T	Rt & Fr	NA	NA	*‘*Minshiro Nekersa*'*; Tumors	Across the regions of Ethiopia [45, 80]	AF, EW, TU, GD, WU, SU, GG, BA, HA (0-2400) [66]
Rosaceae	*Hagenia abyssinica* (Bruce) J.F.Gmel.	Kosso (Amh)	W	T	Rt	De	The root is pounded and mixed with honey, then creamed on the affected part	*‘*Minshiro nekersa*'*	Across the regions of Ethiopia [45]	EW, TU, GD, WU, GJ, WG, SU, AR, BA, HA, KF, SD (2450-3250) [66]
	*Prunus Africana *(Hook. f.) Kalkm	Tikur Enchet (Amh)	W	T	Br	Or	Liquid extracts from *P. Africana *bark is pounded, juiced and taken orally	Benign prostatic hyperplasia, prostate gland hypertrophy	Ghimbi District, West Wollega Zone, Oromia Region [43]	GD, GJ, WG, SU, AR, BA, HA, IL, KF, SD (1700-2500) [66]
	*Rosa abyssinica* Lindley	Kega (Amh)	W	Sb	Rt & Fl	NA	NA	‘Nekersa*'*	Across the regions of Ethiopia [45]	EW, TU, GD, WU, GJ, SU, AR, HA, BA (1900-3300) [66]
Rubiaceae	*Rubia cordifolia *L.	Enchibir (Amh)	W	H	Rt	De	Used in combination with other herbs and applied topically	Internal cancer	Across the regions of Ethiopia [45]	EW, TU, GD, GJ, WU, SU, AR, KF, SD, BA, HA, (1000-2850) [68]
					Rt	Or	The root part is crushed in water for 3 days and taken orally	Lung cancer	Gubalafto District, North Wello Zone, Amhara Region [69]	
Rutaceae	*Fagaropsis angolensis *(Engl.) Dale	Oloncho (Sid)	W	Sb	Fr	NA	NA	Cancer	Dale District, Sidama Zone, SNNP region [52]	TU, WU, GD, GJ, WG, SU, AR, IL, KF, SD, BA, HA (1680-3000) [66]
	*Zanthoxylum *	Gadda (Sid/O ro)	W	T	Lf	Or	The leaves are dried, powdered, cold macerated and drunk	Breast cancer	Hawassa city and Dalle District, Sidama Zone, SNNP Region [52, 55, 56]	GG, BA, HA (900- 1550) [66]
	*chalybeum *Engl.				Rt	NA	NA	Cancer		
Santalaceae	*Osyris quadripartita *Decn.	Queret (Amh)	W	T	Lf	Or	Dried leaf with dried fruit of *Myrsine africana,* is powdered, mixed with water and given orally	Cancer	Fiche District, Oromia Region [54]	EW, TU, GD, WU, GJ, WG, SU, KF, GG, AR, HA, BA, SD (1600-2900) [66]
Sapindaceae	*Dodonaea angustirolia L. f.*	Kitkita (Amh) Ittancha (Sid)	W	Sb	Lf	NA	NA	Cancer	Dale District, Sidama Zone, SNNP Region; Across the regions of Ethiopia [45, 52]	EW, TU, GD, WU, SU, AR, GJ, WG, KF, GG, SD, BA, HA (500-2900) [66]
					NA	NA	NA	Unidentified swellings/ Neck Cancer	Debre Libanos monastery, North Shewa *Zone*, Oromia Region [53]	
Sapotaceae	*Sideroxylon oxyacanthum *Baill.	Bunguude (Sid)	W	Sb	Lf	Or	The leaf part macerated and taken orally	Cancer	Dale District, Sidama Zone, SNNP Region [55]	TU, GD, SU, AR, BA, HA (1250-2800) [68]
Scrophulariaceae	*Craterostigma pumilum *Hochst.	Delashut (Amh)	W	H	Lf & Fl	De	Used in combination with other herbs and applied topically	Cervical and rectal cancer	Across the regions of Ethiopia [45]	GD, EW, SU, HA, BA, WU, GJ, TU (1300-3100) [61]
	*Rhamphicarpa fistulosa* (Hochst.) Benth.	Yeset lib (Amh)	W	H	Fl	Or	Used in combination with other herbs and given orally	Lung cancer	Across the regions of Ethiopia [45]	GD, GJ, WG, KF (800-1650) [61]
Simaroubaceae	*Brucea antidysenterica *J.F. Mill.	Yedega Abalo (Amh)	W	Sb	St & Br	Or	Decoction is drunk	Cancer	Jimma Zone and Bale Zone, Oromia Region [47, 48]	EW, TU, GD, GJ, WG, SU, AR, IL, KF, SD, BA, HA (1650-2800) [66]
		Waginos (Ge'ez)			Lf	De	The leaves are collected and dried, the powder is then applied to the skin	Cancer	Fiche District, North Shewa Zone, Oromia Region [46]	
Solanaceae	*Nicotiana tabacum* L.	Timbaho (Amh)	HG	H	Lf	De	The leaf is pounded and creamed on the swelling	*‘*Nekersa*'*	Across the regions of Ethiopia [45]	EW, TU,GD, SU, WG, KF, GG, SD, HA (300-2400) [61]
	*Solanum gigantum *Jacq.	Tikur Embuay (Amh) Ziza (Kam)	W	Sb	Lf	De	NA	Breast pain	Kembatta Tembaro (KT) Zone, SNNP Region [58]	GD, GJ, WG, IL, KF, SD, BA (1100-2300) [61]
	*Withania somnifera *(L.) Dunal in DC.	Gizawa (Ama)	W	Sb	NA	NA	NA	Cancer	Fiche District, North Shewa Zone, Oromia Region [46]	AF, EW, TU,GD, GJ, WU, SU, KF, GG, SD, HA (600-2700) [61]
Vitaceae	*Cyphostemma adenocaule *(Steud. ex. A. Rich.) Descoings exWild & Drummond	Asserkush (Amh)	W	Cl	Lf	De	The leaf part is warmed up and pasted on the affected area	Swelling by *‘*nekersa*'*	Ada'a District, East Shewa Zone Oromia Region; Across the regions of Ethiopia [31, 45]	EW, TU, GD, GD-GJ, WU, SU, WG, IL, KF, GG, SD, BA, HA (600-2650) [66]
	*Cyphostemma cyphopetalum (Fresen.) Descoings *ex *Wild&Drummond*	Gindosh (Amh) Kelkalo (Oro)	W	Cl	Rt & Fl	NA	NA	*‘*Nekersa*'*	Across the regions of Ethiopia [45]	AF, EW, TU, GD, WU, SU, SU-AR, GJ, WG, GG, SD, BA, HA (250-2800) [66]
*Zygophyllaceae*	*Tribulus terrestris *L.	Ch'amare (Oro)	W	H	Fr	NA	NA	Cancer	Across the regions of Ethiopia [80]	EW, WU, SU, AR, SD, HA (sea level up to 2300) [65]

**Note: PU** ^**§**^ **= part used **(Lf-leaf; Rt=root; Br=bark; Fl; flower; Fr=fruit; Sd=seed; Lx=latex; Sh=shoot; St=stem; WP=whole part);** AR**^**‡**^**=application route **(Or=oral; De=dermal; In=insertion; Ns=nasal); **GF**^Ψ^** = growth forms** (T=tree; Sb=shrub; H=herb; Cl=climber/liana); habitat (W=wild; HG=home garden);** Key**: ^**¥**^local names: Ge'ez: Ge'ezinga; Amh: Amharic; Tig: Tigrigna; Oro: Afaan Oromoo; Sid: Sidamu-afoo; Age: Agewugna; Kam: Kambatissa; Som: Somali; NA= not available. Note: vernacular names of malignancies/cancer are written in small caps, *Italic*, font 10, within single inverted commas throughout the document.

TU: Tigray region above 1000 m contour; AF: Afar region below 1000 m contour to Eritrean border in the east and Harerge border in the south; WU: Welo region above 1000 m contour; GD: Gondar region; WG: Welega region; KF: Kefa region; AR: Arsi region; BA: Bale region; GJ: Gojam region; IL: I1ubabor region; GG: Gamo Gofa region; SD: Sidamo region; HA: Harerge region.

## Appendix

See Table 5.

## Abbreviations

AAU: Addis Ababa University
ABS: Access and benefit sharing
CAM: Complementary and alternative medicine
IARC: International Agency for Research on Cancer
MP/MPs: Medicinal plant(s)
SNNP: Southern Nations, Nationalities and Peoples
TM/TMs: Traditional medicine(s)
WHO: World Health Organization.
